# Effect of Temperature on the Aging rate of Li Ion Battery Operating above Room Temperature

**DOI:** 10.1038/srep12967

**Published:** 2015-08-06

**Authors:** Feng Leng, Cher Ming Tan, Michael Pecht

**Affiliations:** 1 Nanyang Technological University, School of Electrical Electronics Engineering, Blk S2.1, 50 Nanyang Avenue, Singapore 639798, Singapore; 2 TUM CREATE PTE LTD, 1 Create Way, #10-02 Create Tower, Singapore 138602, Singapore; 3 Chang Gung University, Department of Electronics Engineering, Taoyuan, TaiWan 259 Wen-Hwa 1st Road, Kwei-Shan Tao-Yuan, Taiwan, 333, R.O.C; 4 Center for Advanced Life Cycle Engineering (CACE), University of Maryland, College Park, MD 20740, USA; 5 Global Energy Quality And Reliability Technology (G.E.Q.A.R.T). PTE.LTD, Sims Residence, 8 Lorong, 29 Geylang #06-12, Singapore 387882, Singapore

## Abstract

Temperature is known to have a significant impact on the performance, safety, and cycle lifetime of lithium-ion batteries (LiB). However, the comprehensive effects of temperature on the cyclic aging rate of LiB have yet to be found. We use an electrochemistry-based model (ECBE) here to measure the effects on the aging behavior of cycled LiB operating within the temperature range of 25 °C to 55 °C. The increasing degradation rate of the maximum charge storage of LiB during cycling at elevated temperature is found to relate mainly to the degradations at the electrodes, and that the degradation of LCO cathode is larger than graphite anode at elevated temperature. In particular, the formation and modification of the surface films on the electrodes as well as structural/phase changes of the LCO electrode, as reported in the literatures, are found to be the main contributors to the increasing degradation rate of the maximum charge storage of LiB with temperature for the specific operating temperature range. Larger increases in the Warburg elements and cell impedance are also found with cycling at higher temperature, but they do not seriously affect the state of health (SoH) of LiB as shown in this work.

The Lithium-ion batteries (LiB) are a significant technology in today’s global green energy initiative because of their high energy density, long lifetime, reasonable safe operation and affordable cost^1^. They enable the possibility of various types of electric vehicles, space applications and even our everyday handheld electronics. The operating temperature of LiB must be well controlled, as its performance, health, and safety depends on the temperature. Catastrophic failures due to excessive temperature variations especially high temperatures can cause a thermal runaway reaction that ignites a fire and consequently cause an explosion^2^,^3^. Different operating temperatures will also affect the performance of LiB over time at different rates and therefore reduce its lifetime accordingly. Hence, implementation of efficient cooling system is being used for LiB system, but an understanding of the temperature effects on the degradation rate of each component inside LiB will be useful for improving the design of LiB system and extending the LiB’s lifetime. The LiB’s usability can also be expanded if its allowable operation temperature range is extended. Unfortunately, there are only few available literatures on this topic.

Several researches on the effect of temperature on battery degradation of various cell components in LiB have been conducted recently. Markevich *et al.*^4^ studied the degradation of carbon negative electrode at elevated temperature (up to 80 °C); Gabrisch *et al.*^5^ studied the degradation of thermally aged LiCoO2 and LiMn2O4 cathode; Handel *et al.*^6^ studied the thermal aging of electrolytes and investigated the degradation product from electrolyte and the influence of the housing material; Schalkwijk *et al.*^7^ investigated the temperature effect on the degradation at electrode/electrolyte interface, and Ramadass *et al.*^8^. studied the capacity fade of Sony 18650 cells with LiCoO_2_/Graphite electrode materials at room temperature, 45 °C, 50 °C and 55 °C respectively. These work focus on the effect of temperature on individual component in LiB.

The study of temperature on the LiB as entire entity was done by Bodeness *et al.*^9^ and Thomas *et al.*^10^. Using X-ray diffraction (XRD), Nuclear Magnetic Resonance, Scanning Electron Microscopy (SEM) and X-ray Photo-electron Spectroscopy, Bodeness *et al.*^9^ studied the effect of high temperature on the aging process of electrode’s binder, electrodes/electrolyte interfaces, and positive active material. Their results indicated a formation of a binder layer at the surface of the positive electrode and led to a poor Li-re-intercalation. They also showed several changes in the composition of the Solid Electrolyte Interphase (SEI), when the LiBs were cycled at 60 °C and 80 °C and 120 °C respectively and observed the disappearance of carbonate species and the increase of inorganic species at the surface of negative electrode. The modification of the SEI composition layer is proposed as a cause for the capacity loss and impedance increase at high temperature, in addition to the binder on the electrode surface of the positive electrode.

Thomas *et al.*^10^ used scanning electron microscopy (SEM), energy dispersive X-ray analysis (EDX), inductively coupled plasma (ICP), and measurements of electrode thickness to compare the aged and fresh materials of cathodes and anodes, and they showed morphological and structural changes of the electrodes during cycling. Further study on the chemical reactions of degradation was also done using X-ray diffraction(XRD). For the Li_x_Ni_1/3_Co_1/3_O_2_/Li_y_Mn_2_O_4_ blend cathodes and graphite/carbon anodes system (commercial 18650 cells), they showed that the capacity loss is mainly related to the SEI film growth on the anode, and the elevated temperatures accelerate the degradation of the cathode and formation of SEI on anode. The SEI was found to be the main contributor to the increase in the cell internal resistance.

While Bodness *et al.*^9^ and Thomas *et al.*^10^ investigated the aging of various cell components at elevated temperature and provided useful information for the aging of LiB cycled at high temperature, their techniques involve disassembly of battery and complex instrument that is costly and not portable, making their methods not possible to be implemented in some practical applications which required a real time investigation such as those demanded by the battery management system (BMS) of electric vehicle (EV). An *in situ* non-destructive technique (NDT) will be better for the field applications. Also, the respective degree of contribution of each component in LiB to the overall degradation rate of LiB performance at different temperatures and how their respective degradation rates of each component in LiB manifest in term of the electrical performance of LiB are not presented. In fact, while studies on the effect of temperature on the aging of LiB are reported as described earlier, the effect of temperature on the aging rate of LiB is not reported, and this will be investigated in this work.

In this work, the performance degradation rate for each component in an LiB will be examined when it is operating at different temperatures from 25 to 55 °C using the recently developed electrochemistry-based electrical model (ECBE)^11^. Unlike the various reported equivalent circuit models where the models are developed to fit the reported experimental data, ECBE model is developed based on the first principle of electrochemistry, and then convert the corresponding partial differential equations into circuit model. It is verified by the electrochemical impedance spectrometer (EIS) which is the most popular aging characterization tool for different type of LiB cells^11^. EIS employs electrical model to comprehensively understand the different aging behavior in electrochemical system, but its measurement can only be done off-line in frequency-domain with laboratory experiment that is usually inaccessible to field application. Also, complex solution of simultaneous partial differential equations is needed to determine the values of the different components in LiB using EIS^11^. On the other hand, ECBE allows the performance of each component inside LiB be determined real time through its discharging curve nondestructively (i.e., terminal voltage vs. time during discharge), making it suitable for field applications. As ECBE is derived based on the first principles, it can be applied to other cell systems.

The rest of paper is organized as follows: The experimentation is given in Section 2, and the effect of temperature on the aging rate of the maximum charge storage capacity is shown in Section 3.1. In Section 3.2 and 3.3 the effect of temperature on the aging rate of electrodes will be discussed. Section 3.4 shows effect of temperature on the aging rate of the electrolyte. The overview of the temperature effect on aging rates is provided in Section 3.5. Conclusion is given in Section 4.

## Experimentation

**Figure 1:**
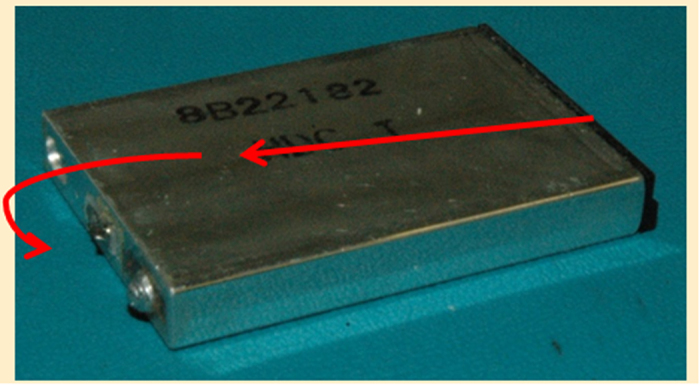
A prismatic cell used in this work. (Picture taken by M. Pecht at the CALCE–University of Maryland).

The LiB studied in this work is a prismatic cell from Sony. Its specifications are shown in Table 1, and Fig. 1 shows a photo of the cells used in this work. The charge and discharge cycles of LiB were performed using an Arbin BT2000 battery testing system in the Center for Advanced Life Cycle Engineering (CALCE) at the University of Maryland, College Park. The fresh cells are firstly discharged to the cut-off voltage of 2.7 V as specified in the battery specification which corresponds to 100% depth of discharge. A standard constant current/constant voltage (CCCV) charging profile with a fixed current rate of 0.5C until the voltage reach 4.2 V, followed by maintaining at 4.2 V until the charging current dropped to below 0.05A, was used for all the cells under study. The cells are rested for 120s after charging for stabilization of its terminal voltage.

**Figure 2:**
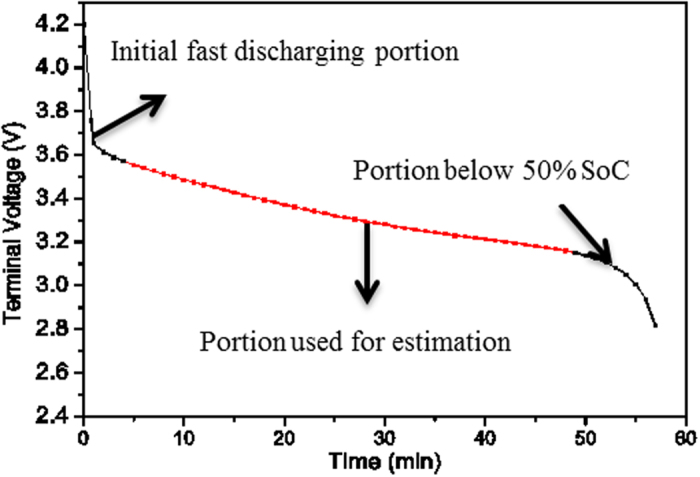
Comparison of an experimental and computed discharge curve (using ECBE model) of a Sony prismatic cell.

**Figure 3:**
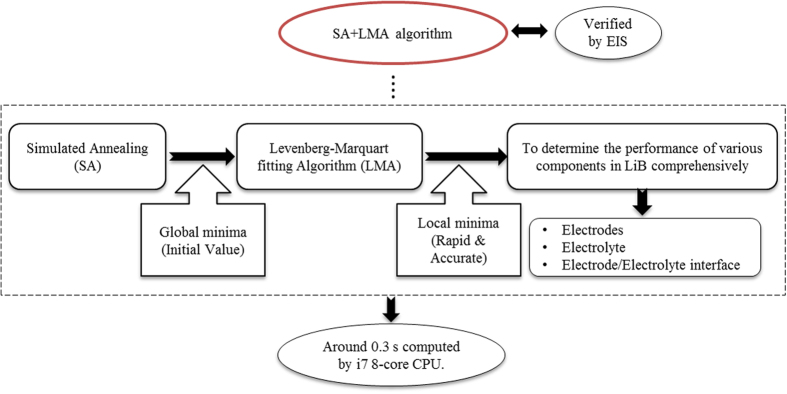
The flow chart of the computation in ECBE model^11^.

For the discharging experiments, four cells were discharged at a constant current of 1C to a cut-off voltage of 2.7 V. One cell was cycled at 25 °C, a separate cell was cycled at 35 °C, a third cell was cycled at 45 °C and fourth cell at 55 °C. The terminal voltage and current of the cells are monitored on-line to ensure safety and recorded every 30s as input parameters for fitting process. The charging/discharging profile was repeated up to 260 cycles. Six particular cycle numbers, namely 1, 50, 100, 150, 200, and 250 were selected to investigate the cycled cells. Figure 2 shows a typical discharge curve of a lithium-ion cell. The red curve (the curve with data points on it) is computed using the ECBE battery model. Simulated Annealing^12^ is used to obtain an approximated global minima, and non-linear regression method, Levenberg-Marquart fitting algorithm (LMA) is employed to provide a rapid and accurate estimate of the local minima^13^,^14^. Figure 3 shows the flow chart of the computation in ECBE model. With these algorithms, a good agreement can be seen between the computed and experimental discharge curves. Note that the ECBE model is valid up to 50% SoC, and the initial fast discharging portion is excluded from the model. Here SoC is computed as *Q*/*Q*_*m*_ where *Q*_*m*_ is the initial maximum charge stored in LiB and it is computed using the ECBE model^11^, and 
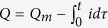
 as evaluated using the Coulomb counting method.

**Figure 4:**
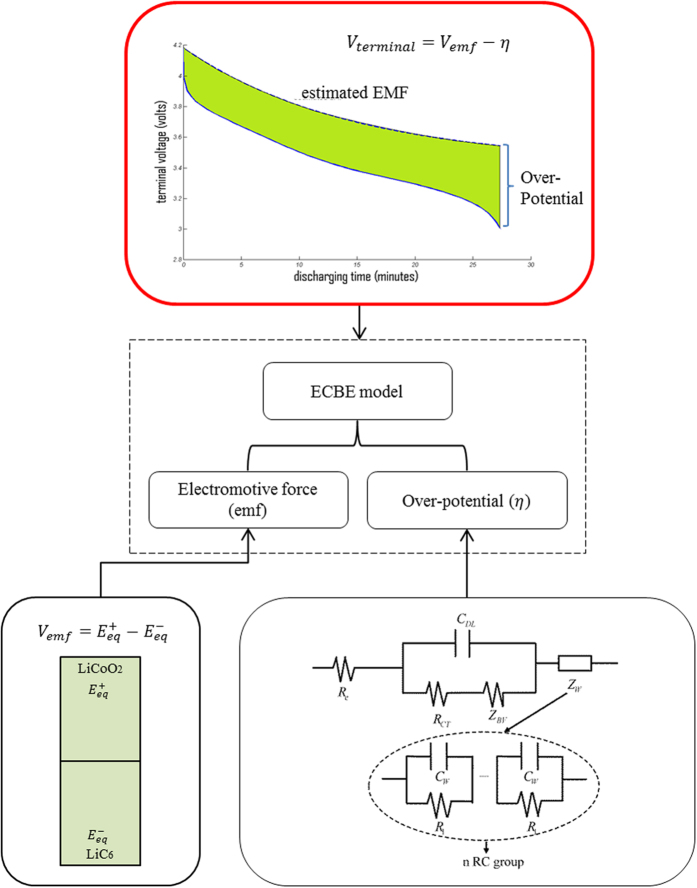
Electrochemistry-based electrical model^11^.

Figure 4 shows the ECBE model, where R_e_ is the combined ohmic resistance of the electrodes and the electrode/electrolyte interface, C_dl_ is the double layer capacitance, R_ct_ is the total charge transfer resistances at the electrodes, K is the charge transfer rate constant at the electrodes embedded in the Butler-Volmer impedance (Z_BV_), and R_n_ and C_w_ are the Warburg element in the electrolyte.

## Results and Analysis

### Effect of temperature on the aging rate of the maximum charge storage capacity

**Figure 5:**
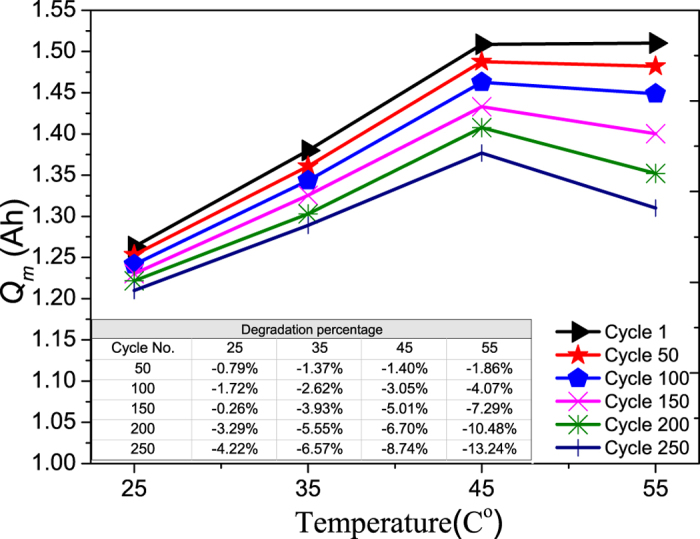
Maximum charge storage capacity as a function of temperature. The maximum charge storage capacity corresponds to different cycle numbers.

By investigating the maximum charge storage capacity (*Q*_*m*_) and the effects of temperature variation from 25 to 55 °C and cyclic aging on the degradation of *Q*_*m*_, valuable results may be generated to aid in the determination of appropriate usage conditions. Figure 5 shows that *Q*_*m*_ gradually decreases with an increasing number of cycles as expected. The degradation mechanisms for this irreversible capacity loss with cycle aging are found to be related to one or more of the following, namely structural changes of the insertion electrode, electrolyte decomposition, active materials dissolution, phase changes in the insertion electrode and passive film formation over electrodes and the current collector surface^15^,^16^.

To be specified, the degradation of an LCO electrode includes its structural changes during cycling, and surface film formation and its subsequent modification on the electrode^17^. For the graphite electrode, the major degradation mechanisms is the formation and growth of the film at solid electrolyte interface (SEI) due to electrolyte decomposition and the solvent co-intercalation process at the graphite electrode^15^,^17^. A closer look at Fig. 4 reveals that the higher the temperature, the larger the *Q*_*m*_, except a drop at 55 °C. Basically, temperature increases LiB’s performance in a short term by increasing its capacity. But it also increases the degradation rate of *Q*_*m*_ as shown in Fig. 5.

The increasing degradation rate of *Q*_*m*_ during cycling with increasing temperature is because the degradation mechanisms of irreversible capacity loss are accelerated by elevated temperature, as reported in many studies^15^,^17^. While the degradation mechanisms of different components in LiB, namely the electrodes, the electrolyte, their interfaces, and the separator, that result in the irreversible capacity loss are known, the order of important of these components’ degradations is unknown. The investigation of this order of important is an objective of this work, and the manifestation of these degradations on the electrical performance of LiB is another objective of this work that has not been explored.

### Effect of temperature on the aging rate of LCO electrode

**Figure 6:**
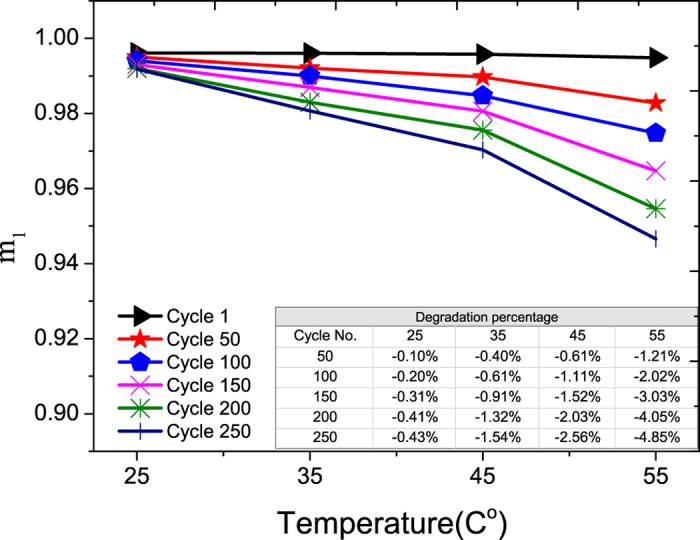
The aging of m_1_ of cobalt-oxide electrode vs. temperature. The percentage degradations vs. cycle number at different temperatures are shown in the inserted Table.

The LCO electrode, which is the cathode during discharging, is made from LiCoO_2_(LCO), the most commonly used material for composite electrodes^18^. Figure 6 shows the degradation of m_1_ of an LCO electrode with cycling at different temperatures ranging from 25 °C to 55 °C. The definition of m_1_ is the effectiveness of the LCO electrode in storing Li-ions^19^.

**Figure 7:**
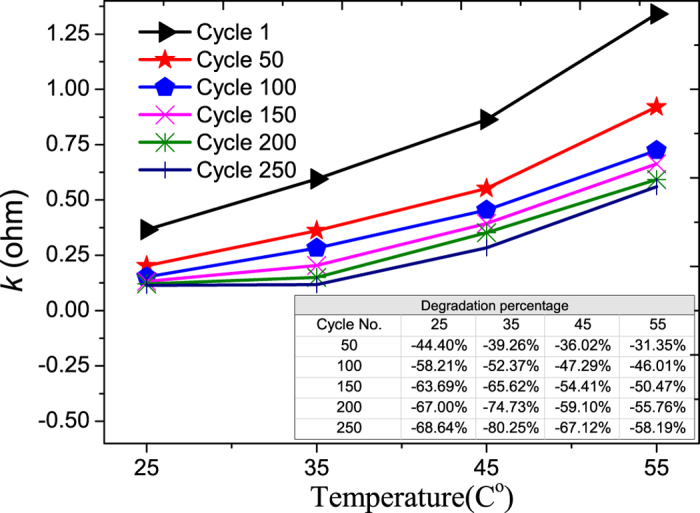
The aging of rate constant vs. temperature. The percentage degradations vs. cycle number at different temperatures are shown in the inserted Table.

**Figure 8:**
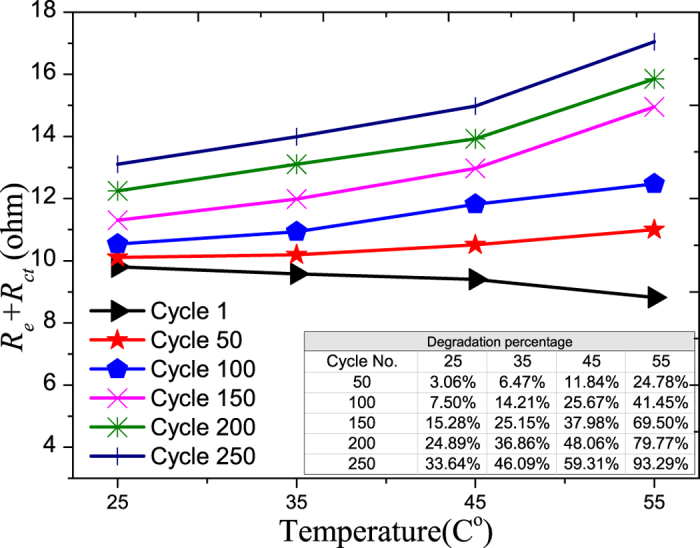
The total resistance of electrodes resistance and electrode/electrolyte resistance aging vs. temperature. The percentage degradations vs. cycle number at different temperatures are shown in the inserted Table.

The degradation of m_1_ can be due to two mechanisms. One is the formation of surface film and its subsequent modification on the electrode, and another one is the structural/phase changes of the electrode. Zhang *et al.*^20^ and Ramadass *et al.*^8^ observed the formation of the surface film as a result of the oxidation at the electrode/electrolyte interface. Maher *et al.*^21^ identified the structural and phase changes of the LCO electrode. The presence of surface film (also called SEI) lowers the reaction rate of both Li^+^ insertion and de-intercalation^20^, and the structural/phase change of the electrode from a hexagonal phase (less stable, but active) to a cubic phase or spinel structure (less active) also reduces the charge transfer rate. Hence, both mechanisms result in a decrease in the charge transfer rate (K) with cycling. This charge transfer rate shows the rate of Li+ transport as it goes from electrode to electrolyte and from electrolyte to electrode^22^. The above-mentioned two mechanisms will reduce the transport rate, and this is indeed observed in Fig. 7. Both will also increase the electrode’s impedance, and again this is observed in Fig. 8.

In fact, two different degradation mechanisms are observed in Fig. 7, namely the large initial drop in K value, followed by a slower decrease in the K value after 100 cycles. For the two mechanisms mentioned above, our current analysis cannot identify which of the two will occur first. On the other hand, a closer examination of Fig. 7 shows that the rate of decrease of the K value after 100 cycles is not significantly affect by temperature when the temperature range is 35–55 °C. This information could shed some light on the identification of the dominant mechanism in the later part of the cycling aging.

As the decrease in the value of K and the increase of the electrode impedance are due to the same mechanisms, their dependency on temperature are expected to be the same, and this can be seen in Fig. 7 and Fig. 8.

### Effect of temperature on the aging rate of graphite electrode

**Figure 9:**
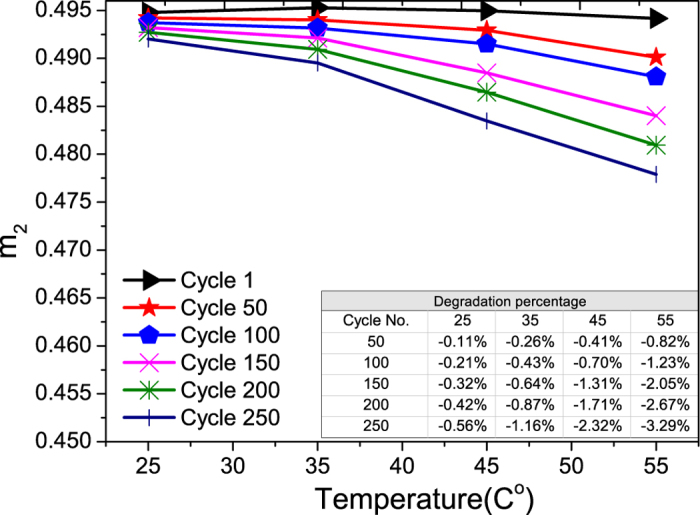
The aging of m_2_ of graphite electrode vs. temperature. The percentage degradations vs. cycle number at different temperatures are shown in the inserted Table.

Graphite is the most important anode electrode material in LiB as it has high capacity, flat potential profile and possess several advantages such as low cost, long life cycle, low volume expansion and safety^23^,^24^. Figure 9 shows the degradation of the m_2_ of the graphite electrode with cycling under various temperatures. The m_2_ in the ECBE model represents the effectiveness of the graphite electrode in providing its stored Li-ions^19^.

The degradation of m_2_ is found mainly due to the formation of the SEI and its growth on the surface of the graphite electrode with cycling^7^. This SEI is developed through reductive electrolyte decomposition accompanied by irreversible consumption of lithium ions that leads to the irreversible capacity loss with the eventual release of gaseous products. Since this SEI layer is not completely permeable for lithium ions, the amount of Li-ions that can be provided from this electrode reduces with the continuous growth of the SEI at the graphite electrode^25^. This SEI will also result in a decrease in the charge transfer rate (K) and an increase in the cell impedance, as in the case of an LCO electrode. Another possible mechanism for the aging of the graphite electrode is that the solvent can co-intercalate into the carbon, causing the exfoliation of the carbon and the subsequent expansion of the carbon particles which form a ternary graphite intercalation compounds (GIC). The development of the (GIC) leads to the loss of active material, and it will also contribute to the irreversible capacity loss^15^. However, development of the GIC will not affect the charge transfer rate^15^,^20^.

If LiB is operating at a higher temperature, the growth rate of SEI will be increased, and this will impede the delivery of the Li-ions from the graphite electrode. Higher temperature will also enhance the formation of GIC. Therefore, both mechanisms will cause a larger degradation of m_2_ at higher operating temperatures as shown in Fig. 9, and this finding is consistent with Thomas *et al.*^10^.

A careful examination of the inserted Tables in Fig. 6 and Fig. 9 reveal that the degradation rates for both electrodes during cycling are quite similar at 25 °C. But the degradation rate of LCO electrode is affected more when the temperature is above 25 °C. This implies that the rate of degradation of the LCO electrode is more temperature dependent than that of the graphite electrode, and this can also be seen from the larger increase in the slope of degradation for the LCO electrode.

The degradation rates of the electrodes increase with temperature as discussed earlier. A larger jump in the cell resistance in the early stages of cycling is observed at high operating temperatures, as can be seen from 0 to 50 cycles in Fig. 8. This is believed to be due to the increasing rate of SEI formation at the electrodes at higher temperature where Schalkwijk *et al.*^7^ have elaborated on the mechanism of SEI formation at different temperatures. Beyond 50 cycles, binder decomposition, oxidation of the conductive agent, and corrosion of the current collector will also contribute to the impedance increase, causing another large increase in the resistance at high operating temperature, as can be seen from 100 to 150 cycles in Fig. 8^17^.

### Effect of temperature on the aging rate of the electrolyte

The aging of the electrolyte can be analyzed through the change in the Warburg element. This Warburg element models the electrolyte as a dielectric of a parallel plate capacitor with the two electrodes as the two plates of a capacitor. It models the electrolyte system as a series of R_w_ and C_w_ where R_w_ is relate to the resistance of the electrolyte and C_w_ is related to the capacitance of the equivalent parallel plate capacitor.

**Figure 10:**
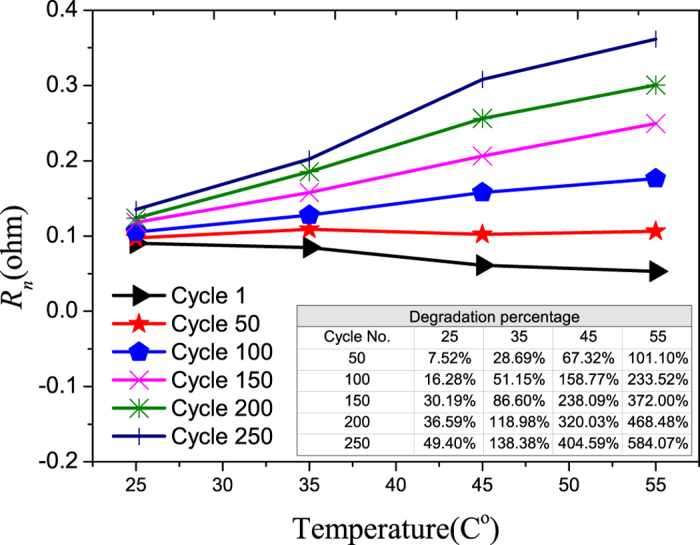
The aging of Warburg element resistance vs. temperature. The percentage degradations vs. cycle number at different temperatures are shown in the inserted Table.

As temperature increases from 25 °C to 55 °C, the diffusivity of active Li-ions in the electrolyte increases^26^, and the Li-ion concentrations that flows through the electrolyte also increases^19^ due to the increase in Q_m_ as a result of the enhanced electrochemical reduction-oxidation (redox) at anode and cathode at elevated temperature^27^,^28^, thus a decrease in the resistance of the electrolyte is expected when the cell is initially cycled, as observed in Fig. 10.

**Figure 11:**
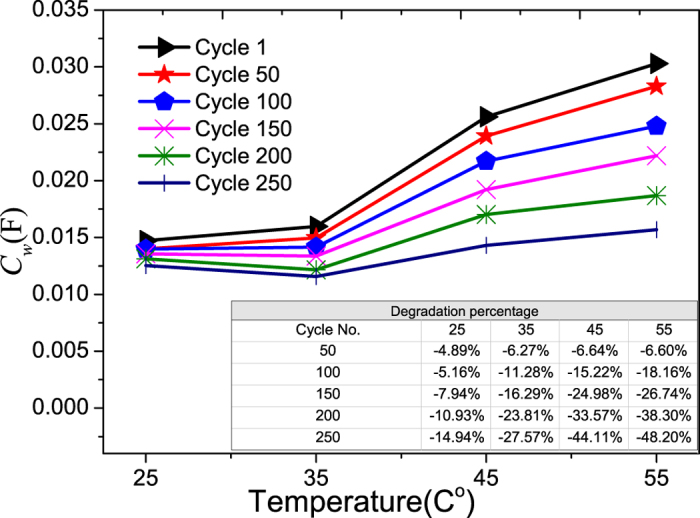
The aging of Warburg element capacitance vs. temperature. The percentage degradations vs. cycle number at different temperatures are shown in the inserted Table.

On the other hand, the Warburg element capacitance increases with temperature as shown in Fig. 11. This can be explained by the increasing number of ionic charge stored (due to the increase in Q_m_) in the two electrodes, i.e. 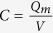, and for a given V, which is the voltage across the two terminals of LiB, increase in Q_m_ will result in an increase in C.

The degradation rate of R_n_ (i.e., the increase in the value of R_n_) due to cycling is larger at higher temperatures. This may be due to the increasing degradation rate of the maximum charge storage capacity with cycling at higher temperatures. The increasing degradation rate of separators at higher temperatures is also a possible reason for the increase in R_n_^10^.

The decrease in the Warburg element capacitance with cycling can be seen in Fig. 11. One plausible cause is the formation of SEI on the electrodes and separator that decrease the available surface of the active materials during cycling, i.e. the effective area of the equivalent parallel capacitor is reduced. Another possible reason is due to the formation of SEI layer which modifies the capacitor model of the electrolyte into two capacitors in series, where one of them is having the electrolyte as dielectric, and another one is having the SEI as its dielectric. If the relative permittivity of the electrolyte is ε_1_, and that of the SEI film is ε_2_, the effective capacitance will be 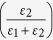, which is always less than 1. As temperature increases the SEI film will also grow faster and thicker, which directly correspond to the decreasing rate of capacitance when cycling and temperature increase.

**Figure 12:**
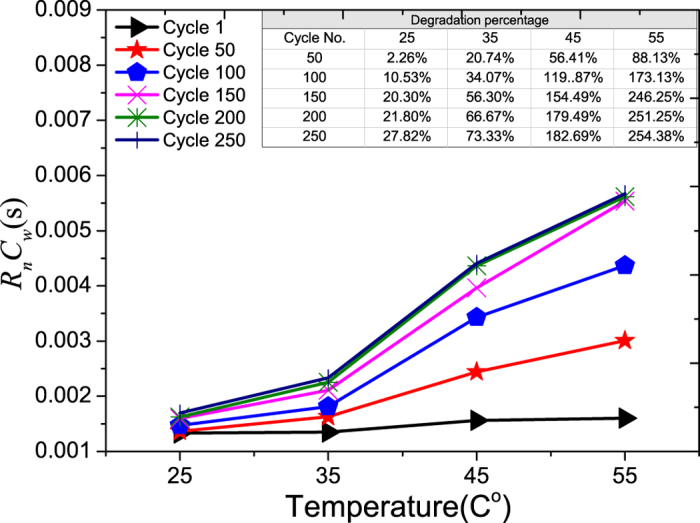
The aging of Warburg RC time constant vs. temperature. The percentage degradations vs. cycle number at different temperatures are shown in the inserted Table.

Multiplication of R_n_C_w_ will result in a graph shown in Fig. 12, and one can see that increasing the temperature will increase the rate of RC, and this implies that the response to the change in current deliver from LiB will become slower at high temperature.

### Overview of the temperature effect on aging rates

From the above analysis, it was found that higher temperature will increase the degradation rates of all the components in a LiB, and this is consistence with the work of Thomas *et al.*^10^.Careful examination of the Tables inserted in the Figures that show the degradation as a percentage of each component in a LiB reveals that temperature has the largest impact on the degradation rate of the Warburg element with cycling, and followed by the cell impedance. The degradation rate of the charge transfer rate is less impacted by the operating temperature for the temperature operating range considered here.

As the operating temperature of LiB changes from 25 to 55 °C, the degradation rate of maximum charge storage after 260 cycles is found to increase from 4.22% to 13.24%. At the component level, for the same change in the operating temperature, the degradation rate of the Warburg element resistance after 260 cycles increases from 49.40% and 584.07% (Fig. 10) which is the highest change; and that for the cell impedance ranks second, increasing from 33.64% to 93.29% (Fig. 8). As for the charge transfer rate, the change in its degradation rate decreases from 68.64% to 56.19% (Fig. 7).

From the change in the degradation of the various components, and compare with the change in the degradation of Q_m_, which also represent the degradation rate of the state of health (SoH) of LiB, we can conclude that SoH degradation is not affected much by the degradation of the Warburg element and cell impedance, as large changes in their values can only result in a small change in the Q_m_. This seems to contradict some studies^8^,^20^ which state that higher cell impedance is the cause of charge capacity loss. The discrepancy may be explained as follows.

In most cases, Q_m_ is determined via Coulomb counting method where Q_m_ is represented by integrating the discharge current with respect to time until the LiB is fully discharged which correspond to the terminal voltage of around 3–2.5 V, depending on the battery type. It is observed that with higher cell impedance, the Q_m_ so determined will be smaller, and this was attributed to the loss of energy to the cell impedance resulting in lesser Q_m_ to flow out of the LiB for integration^15^,^28^. The loss of energy is in the form of *i*^2^*R*. However, this would mean that the temperature of the cell increase slightly, and since Q_m_ increase with the cell temperature as we have observed earlier^19^, such explanation is questionable. Furthermore, as it is evident that the determined Q_m_ (commonly called Q_d_, the discharge capacity) using the Coulomb counting method is higher when the discharging current is smaller^28^, and since lower discharging current will implies lower energy loss, the increase in the cell temperature will be smaller, hence Q_m_ should be decreased with smaller discharging current as compared to larger discharging current, and this contradict to the experimental observation.

We proposed that the observation of lower Q_m_ for cell with higher impedance may be due to larger internal voltage drop in LiB. Thus, when the external voltage of LiB is dropped to 2.7 V where it is set as the voltage where all the stored charges are discharged, the actual voltage within the LiB is actually higher than 2.7 V, and hence not all the stored charges in the cell are extracted to the external circuit. Consequently, the determined Q_m_ is smaller than the actual Q_m_ in the cell. With this explanation, when the discharging current is smaller, the internal voltage drop in the LiB will also be smaller for a given cell impedance, and thus the external 2.7 V will be closer to the actual voltage within the LiB, implying that the remaining stored charges in LiB will be lesser when the discharging is stopped 2.7 V, thus the determined Q_m_ is higher. In other words, the observation of higher cell impedance cause a lower Q_m_ is a measurement artefact instead of cause and effect relationship.

**Figure 13:**
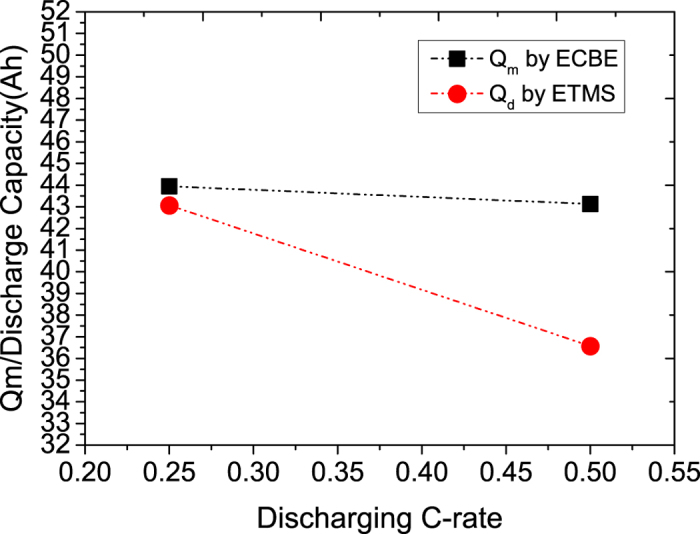
Discharge capacity (Q_d_) measured by ETMS vs. maximum charge storage capacity (Q_m_) estimated by ECBE.

On the other hand, the determination of Q_m_ in this work is computed from the ECBE model, and hence the effect of internal voltage drop due to cell impedance will not affect our calculation. Figure 13 shows comparison of the Q_m_ determined using the Coulomb counting method at different discharging currents versus the Q_m_ determined using the ECBE model. It can be inferred that the trend of Q_d_ using the Coulomb counting method is very similar to the value determined using the ECBE model when the discharging current is small, indicating that the Q_m_ from ECBE model is close to the actual charge capacity of the LiB. The slight reduction in Q_m_ determined from the ECBE model, as seen in Fig. 13, is due to the excessive charges reaching the negative electrode per unit time that render an inefficient storage of charges in the electrode as reported^11^.

From the above analysis, we can see that when SoH is degraded significantly, where *SoH* = *Q*_*m*_(*current*)/*Q*_*m*_(*fresh*), the cell impedance would have been increased very significantly, and this will result in an increase in Joule heating of the cell and lead to thermal runaway and thus possibly create a fire hazard. Therefore, a limited allowable SoH degradation value should be imposed for safety consideration.

**Figure 14:**
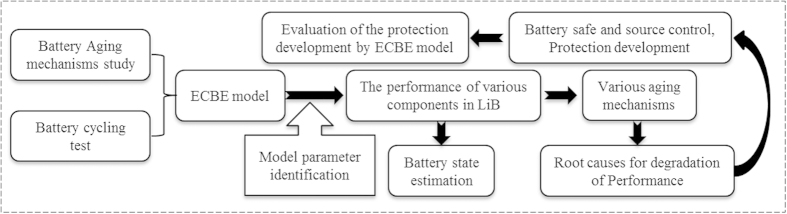
An advanced battery management technology with prognosis and diagnosis capability.

Also, as our method is able to detect the degradation of SoH in real time and simple measurement, it will be useful for LiB prognostics and diagnostics as illustrated in Fig. 14. This information will also allow the determination of remaining useful life of LiB which will be reported in our future work.

## Conclusion

Temperature is an important factor that affects the health and safe operation of LiBs. In this study, we developed a non-destructive *in-situ* approach through ECBE model to detect and characterize the effect of temperature on cycling aging rate in LiB when it is operating in the temperature range of 25 to 55 °C. The performance degradation rate of each component inside the LiB due to cycling aging at different temperatures was determined.

From our analysis, we can see that increasing the operating temperature increases the degradation rates of all components in the LiB which include maximum charge storage capacity, the effectiveness of the LCO electrode in storing Li-ions, charge transfer rate constant, effectiveness of the graphite electrode in providing its stored Li-ions, total resistance of electrode resistance and electrode/electrolyte resistance, Warburg element resistance, Warburg element capacitance and Warburg RC time constant. The increase in the degradation rates of the Warburg element and cell impedance are particularly sensitive to the operating temperature. We also showed that the increase in the degradation rate of irreversible capacity loss of LiB (i.e. SoH) with temperature is due mainly to the formation and modification of the surface films on the electrode and to the structural/phase changes of the LCO electrode.

As low temperature operation below 25 °C can also be important, investigation on the low temperature on the aging rate could also shed light on the good design of LiB for low temperature operation. However, due to the unavailability of the test facility, such work can only be performed as future work.

ECBE model is capable of comprehensively identify the various aging causes through *in-situ* real time characterization. With this information given to battery management system, online monitor the current health status of battery is possible, and one can then reduce the battery loads, namely the voltage, current and temperature according to its health, preventing it from overloading, and ensuring the safety and extend the life of a battery pack.

## Additional Information

**How to cite this article**: Leng, F. *et al.* Effect of Temperature on the Aging rate of Li Ion Battery Operating above Room Temperature. *Sci. Rep.* **5**, 12967; doi: 10.1038/srep12967 (2015).

## Figures and Tables

**Table 1:** Sony Prismatic LiB Specification is provided to distributors by manufacturers.

Battery	Characteristics
Series	Prismatic cell
Chemical System	LCO
Nominal Voltage	3.6 V
Capacity	1,350 mAh Typical
Charging Condition	CVCC 4.2 V max.0.5 C-rate (675 mA), 50 mA cut-off 25 °C
Discharging Condition	CONSTANT CURRENT, 2.7 V cut-off 25 °C
**Dimensions (mm)**	6.6 × 33.8 × 50
Approx.Weight	28(g)
